# Prevention of Osteoporosis in SAMP6 Mice by Rikkunshi-To: Japanese Kampo Medicine

**DOI:** 10.3390/life15040557

**Published:** 2025-03-29

**Authors:** Kouichi Yamamoto, Keiko Yamamoto

**Affiliations:** Department of Radiological Sciences, Faculty of Health Sciences, Morinomiya University of Medical Sciences, 1-26-16 Nanko-Kita, Suminoe-Ku, Osaka 559-8611, Japan

**Keywords:** cortical bone, femur, osteoporosis, Rikkunshi-To, SAMP6 mice, X-ray image

## Abstract

Osteoporosis can increase the risk of fracture in elderly patients, and insufficient control affects quality of life. Rikkunshi-To (RKT) has been prescribed for elderly patients to improve gastrointestinal function. We postulated that RKT has preventive potential for the development of osteoporosis. Thus, we developed a simple method to evaluate osteoporosis using a continuous series of X-ray images of femurs in mice, and investigated the effects of RKT on the development of osteoporosis in these mice. Male senescence-accelerated mouse strain P6 (SAMP6) mice, a model of senile osteoporosis in humans, were fed diets with or without RKT (1%). We collected X-ray images of the whole body of each mouse weekly and measured the ratio of cortical thickness of the femur (C/F index). The C/F index in SAMP6 mice fed the normal diet was increased between 50 and 80 days old, but it was significantly decreased after 120 days old. On the other hand, the C/F index in SAMP6 mice fed the RKT diet was increased between 50 and 80 days old; however, it remained unchanged throughout the experimental period. We also confirmed that the C/F index in SAMP6 mice fed the RKT diet suddenly decreased on the replacement of the RKT diet with a normal diet, suggesting that we can collect data related to a series of continuous changes in bone mass, and that RKT is useful for the prevention of osteoporosis.

## 1. Introduction

Osteoporosis is a common metabolic bone disorder in the elderly, usually resulting in bone pain and an increased risk of fragility fracture. Osteoporosis is often linked to an imbalance in activity of basic multicellular units (BMU) that is responsible for bone turnover and consists of the osteoclasts, osteoblasts, osteocytes, and lining cells [1]. The global prevalence of osteoporosis and osteopenia is about 20 and 40%, respectively [2]. The prevalence is known to be higher in developing than developed countries; thus, the detection and prevention of osteoporosis and osteopenia should be possible with reasonable and simple methods.

Bisphosphonates, RANK ligand inhibitors, sclerostin inhibitor, and parathyroid hormone are clinically used for treatment [3,4,5]. These drugs have marked therapeutic effects against osteoporosis, but it is impossible to readily use them, since they are prohibitively expensive. Also, insufficient control affects the ability to continue therapy. Rikkunshi-To (RKT), a traditional herbal Japanese medicine, acts as a prokinetic agent to improve gastrointestinal (GI) function via the potentiation of ghrelin secretion. Since it is not expensive, it has been widely prescribed for patients with various GI symptoms, such as anorexia, nausea, and vomiting; also, it is often used for gastroesophageal reflux disease (GERD) in the elderly [6,7]. Chiba et al. reported that hesperidin, one of the ingredients of RKT, inhibits bone loss in androgen-deficient male mice or ovariectomized (OVX) female mice [8]; thus, we postulated that RKT, which has been widely used to treat GI disorder, could be used as an agent for the treatment or prevention of osteoporosis, and that RKT would achieve status as a repositioned drug and be potentially useful in geriatric medicine.

The gold standard for the detection of osteoporosis and osteopenia is the measurement of bone BMD by dual-energy X-ray absorptiometry (DXA) [9]. However, DXA requires specific, expensive equipment, as well as well-trained technicians. Furthermore, frequent examinations in a short period result in a higher cumulative radiation dose, which could potentially raise the risks of developing cancer and radiation-related health problems. To overcome such problems, we previously developed a simple method for determining the BMD of patients from the clavicle on chest X-ray by graphical analysis using patient data [10,11]. Through a series studies, we concluded that it is necessary to acquire data on bone mass decrease from healthy individuals and follow-up with the same individuals in order to capture the characteristics of osteoporosis development. Previous studies reported that the senescence-accelerated mouse strain P6 (SAMP6) is a model of senile osteoporosis, which possesses many features of senile osteoporosis noted in humans [12,13,14]. Chen et al. reported that bone mineral density (BMD), femoral weight, femoral calcium, and phosphorus levels were significantly reduced in SAMP6 at 5 months of age, compared with age-matched normal mice, SAMR1 [14]. From these, we collected time-series and continuously changing data on bone mass decreases using X-ray images obtained from SAMP6 mice until mice were about 160 days old in order to develop a method to detect osteoporosis and osteopenia, and to evaluate the therapeutic and preventive effects of RKT on bone mass decreases in SAMP6 mice.

## 2. Material and Methods

### 2.1. Animals

All experiments were approved by the Animal Care Committee of Morinomiya University of Medical Sciences (2021A002, 2023A005). Male SAMP6 and SAMR1 mice were obtained from Japan SLC (Shizuoka, Japan). Among the SAM strains, SAMR1 mice do not show senescence-related disorders, including osteoporosis and osteopenia. Since mice of this strain present a normal aging pattern [15], they have been most frequently used as a control strain.

### 2.2. General Procedure

**Figure 1:**
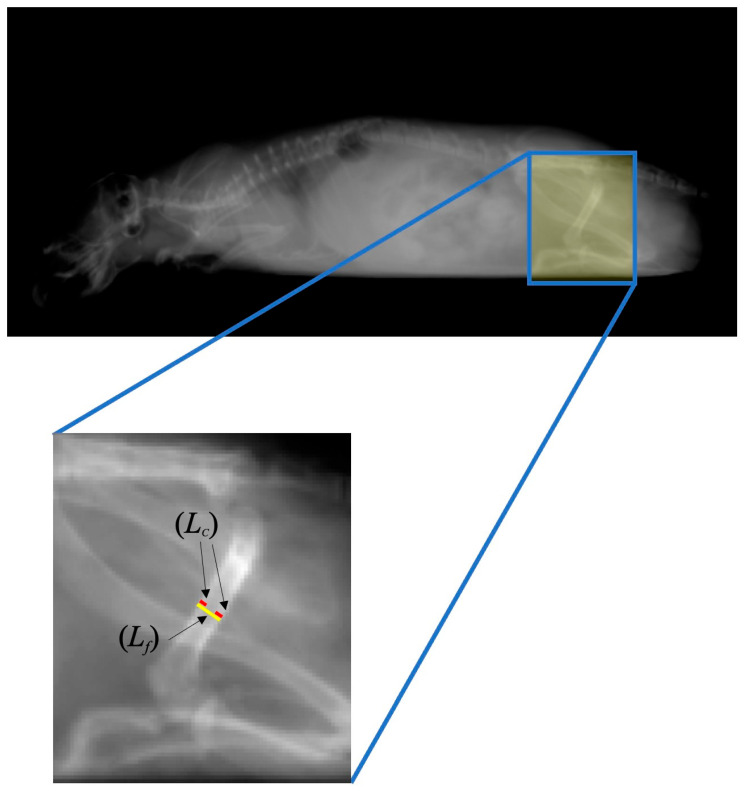
Method to calculate the ratio of cortical thickness of the femur (C/F index). After obtaining X-ray images of a mouse femur, the axial lengths of cortical bone in the femur (*L_c_*: red line) and entire femur (*L_f_*: yellow line) in the captured images were measured, and then the ratio between *L_c_* and *L_f_* was calculated by using Equation (1).

**Figure 2:**
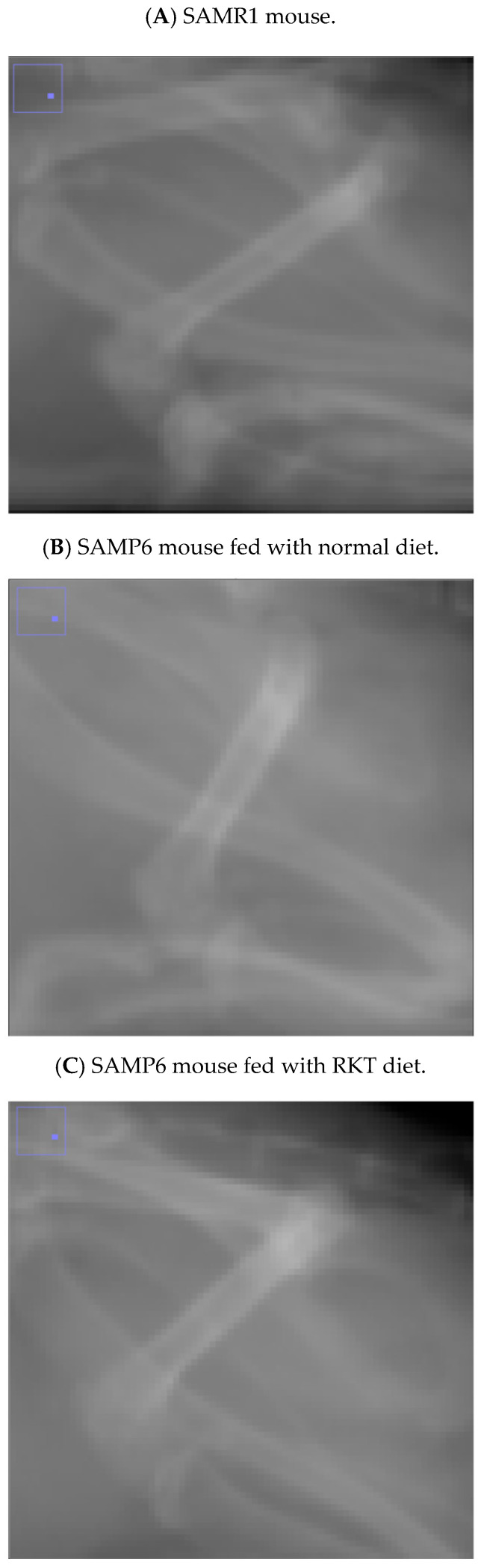
The X-ray photographs show representative femur images in a 150-day-old (**A**) SAMR1 mouse, (**B**) a SAM P6 mouse fed the normal diet, and a (**C**) SAMP6 mouse fed the Rikkunshi-To (RKT) diet.

Approximately 20-day-old mice were housed in individual home cages (14 cm × 21 cm × 12 cm) in a room with a regular light/dark cycle (lights on 600 h–1800 h) at a constant temperature (approximately 24 °C) and humidity (approximately 50%). They were allowed free access to tap water and commercially available standard chow (MF, Oriental Yeast, Osaka, Japan). The mice were adapted to the experimental environment until they were around 40 days old. On the day of image acquisition, mice intraperitoneally (i.p.) received medetomidine hydrochloride (0.6 mg/kg; Dorbene^®^ vet, Kyoritsu Seiyaku Co., Ltd., Tokyo, Japan) for sedation, and total body radiographs were taken using an X-ray machine (NAOMi-CT, RF Co., Ltd., Nagano, Japan) with 50 kVp and 10 mA. The focus-to-image receptor distance was 40 cm and the exposure time used was 0.4 s. X-ray images were captured using ImageJ analysis software (Version 1.53k: developed by Wayne Rasbands, National Institutes of Health, Bethesda, MD, USA), and then the ratio of the cortical thickness of the femur (C/F index) was calculated by following Equation (1) (see Figure 1 and Figure 2).

(1) $$C/F\;index=\frac{axial\;length\;of\;cortical\;bone\;of\;{rat}^{\prime }s\;femur\left(L_{c}\right)}{axial\;length\;of\;{rat}^{\prime }s\;entire\;femur\left(L_{f}\right)}\times 100$$

After image acquisition and calculation of the C/F index, mice i.p. received atipamezole hydrochloride (1.0 mg/kg; Atipame^®^ vet, Kyoritsu Seiyaku Co., Ltd., Tokyo, Japan) to reverse the sedative effect of medetomidine. Image acquisition and evaluation of the C/F index were repeated every 7 days until the mice were about 160 days old, and the daily average of the C/F index over a period of 10 days was analyzed. There were five to eleven mice in each group.

After the experiments had been completed, the mice were euthanized by i.p. injection of excess sodium pentobarbital (150 mg/kg). All drugs were purchased through a pharmaceutical agency (Katayama Chemical Industries, Osaka, Japan) and prepared immediately before use. The indicated doses are those of the free base.

### 2.3. Effects of RKT on Osteoporosis and Osteopenia in SAMP6 Mice

The same experimental method as that described in Section 2.1 was used, except that SAMP6 mice were fed a diet containing RKT throughout the experimental period. The methods used to prepare RKT chow pellets and to administer RKT were those described in our previous studies [16,17]. Powdered standard diet (MF) was mixed with 1% (*w*/*w*) RKT distilled water to form pellets of a similar size to commercially available chow pellets. These chow pellets (Lot: 240238) were dried at 80 °C for 3 h and at room temperature for 1 h. The dose of RKT administered in this study was calculated from the human clinical dose and human-equivalent dose in mice. There were nine mice in a group.

After completing the evaluation of the C/F index on the 160th day, the RKT diet was replaced with a normal diet. The SAMP6 mice were adapted to the experimental environment for 20 days. Then, the C/F index was evaluated at 180 days old. After experiments had been completed, the mice were euthanized by i.p. injection of excess sodium pentobarbital (150 mg/kg).

### 2.4. Rikkunshi-To

We obtained RKT from Tsumura & Co. (Tokyo, Japan, Lot. 2240043010) as a dried powder extract with a similar quality grade to that for medical use. This extract was obtained by spray-drying a hot water extract of the following mixture of crude drugs: Atractylodes lancea rhizome (*Atractylodis lanceae rhizoma*) 4.0 g, Ginseng (*Ginseng radix*) 4.0 g, Pinellia Tuber (*Pinelliae tuber*) 4.0 g, Poria Sclerotium (*Poria*) 4.0 g, Jujube (*Ziziphi fructus*) 2.0 g, Citrus Unshiu Peel (*Citri Unshiu Pericarpium*) 2.0 g, Glycyrrhiza (*Glycyrrhizae radix*) 1.0 g, and Ginger (*Zingiberis rhizoma*) 0.5 g.

### 2.5. Analysis of HU of the Fourth Lumbar Spine of Mice

**Figure 3:**
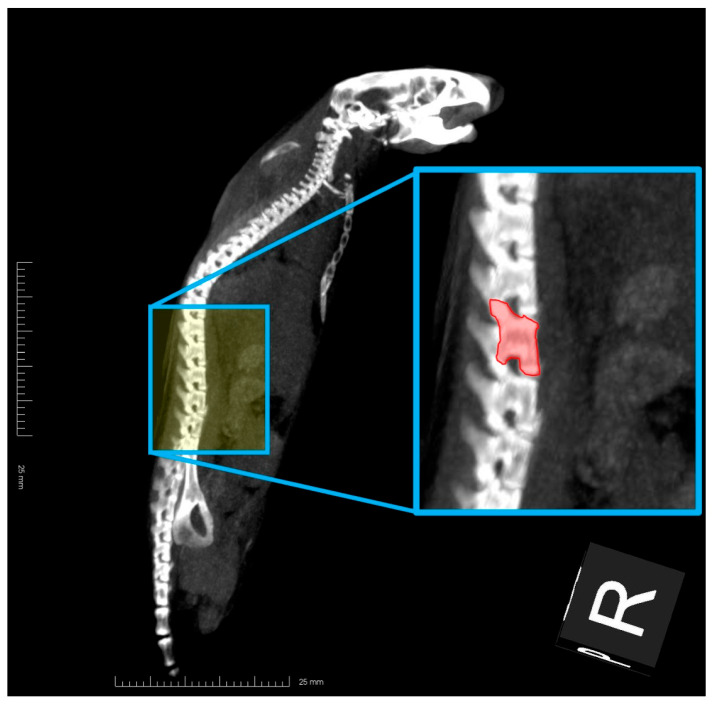
Method of calculating the Hounsfield units (HU) of the lumbar spine of mice determined by standardized region of interest (ROI) area size (HUL4 index). After obtaining X-ray CT images of a mouse, a sagittal reconstruction view of the spine was made. After capturing the images, the mean HU within the ROI that was placed in the fourth lumbar vertebral body (an area that is completely red) and the area value of ROI were obtained, and these HU indexes were determined by standardized ROI area size using Equation (2).

Since it is known that the BMD of the lumbar spine is closely correlated with Hounsfield units (HU) on X-ray CT [18], HU have been used for the diagnosis of osteoporosis. To verify the evaluation of osteoporosis and osteopenia based on the C/F index, we measured the HU of the lumbar spine of mice. Total-body CT images of SAMR1 or SAMP6 mice fed the diet with or without 1% RKT were acquired using an X-ray machine (NAOMi-CT, RF Co., Ltd.) with 50 kVp and 5 mA, and a sagittal reconstruction view of the spine (slice thickness: 5 mm) was made. After capture using Image J, mean HU within the region of interest (ROI) placed in the fourth lumbar vertebral body (L4), and the area of ROI was obtained (please see Figure 3), and the HUL4 index was calculated by following Equation (2).

(2) $$HUL4\;index=\frac{mean\;Hounsfield\;unit\;of\;region\;of\;interest}{area\;value\;of\;ROI\;placed\;in\;the\;fourth\;lumbar\;vertebra}$$

CT image acquisition and evaluation of the HUL4 index were repeated every month until the mice were 160 days old.

### 2.6. Statistical Analysis

The data are expressed as the mean values ± S.D. All differences were analyzed using one- or two-way analysis of variance (ANOVA) followed by post hoc Bonferroni’s multiple comparison tests, as appropriate. *p*-values less than 0.05 were considered significant. Sample size and power calculations were also conducted. All statistical analyses were performed using GraphPad Prism 10.4.1 (GraphPad Software, La Jolla, CA, USA).

Two independent examiners were assigned to analyze the C/F index and HUL4 index. The two examiners conducted their analysis of all of the mice. Pearson correlation analysis was conducted to validate the results of the C/F index and HUL4 index between the two examiners.

## 3. Results

### 3.1. C/F Index of SAMR1 Mice

**Figure 4:**
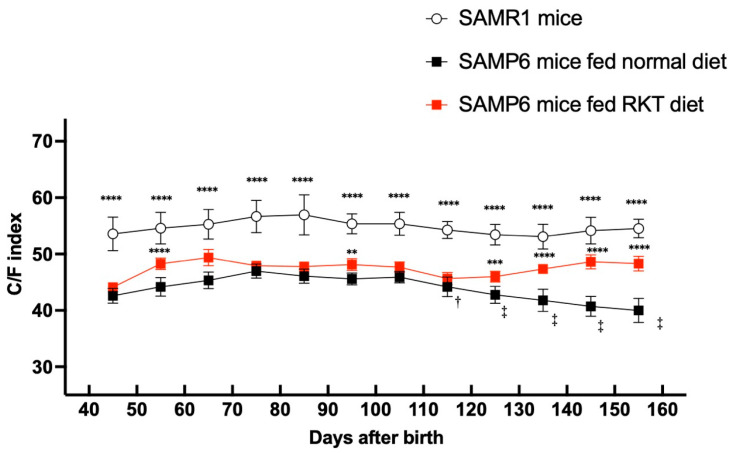
The time-course profile of ten-daily changes in the C/F index of SAMR1 mice, SAMP6 mice fed the normal diet, and SAMP6 mice fed the RKT diet. The X-ray image acquisition was repeated every 7 days from the age of about 40 to 160 days old, and the ten-daily average of the C/F index was analyzed. Points and bars represent the mean ± S.D. of the ten-daily C/F index. The data were analyzed for significant differences using two-way analysis of variance (ANOVA), followed by post hoc Bonferroni’s multiple comparison tests. ** *p* < 0.01, *** *p* < 0.001, and **** *p* < 0.0001 vs. SAMP6 mice fed the normal diet. † *p* < 0.05, ‡ *p* < 0.01 vs. each respective peak value. There were nine to eleven mice in each experimental group.

As shown in Figure 4, there was an upward trend in the C/F index of SAMR1 control mice. The value at the beginning of this study was 53.6 ± 2.9, and it increased along with mouse growth. The value peaked when mice were 80 days old (57.0 ± 3.6), and remained unchanged thereafter. The Pearson correlation found between the two examiners who measured the C/F index was 0.9971 (*p* < 0.05).

### 3.2. C/F Index of SAMP6 Mice Fed Normal Diet

As shown in Figure 4, the C/F index at the beginning of this study was 42.6 ± 1.3, and it increased along with growth in SAMP6 mice fed the normal diet. Similarly to SAMR1 mice, the value peaked (47.0 ± 1.3) when the mice were 80 days old, and then it remained mostly constant for 40 days. However, the value gradually but significantly dropped when the mice reached 120 days old, and the value at 160 days old was less than 83 percent of the peak value (39.3 ± 1.8). The C/F index in SAMP6 mice fed the normal diet remained significantly lower than that in SAMR1 mice over the entire experimental period. The Pearson correlation found between the two examiners’ who measured the C/F index was 0.9996 (*p* < 0.05).

### 3.3. C/F Index of SAMP6 Mice Fed RKT Diet

**Figure 5:**
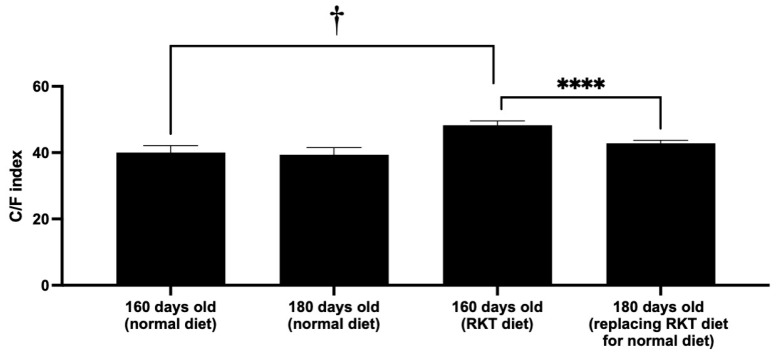
Effect of RKT on changes in the C/F index of SAMP6 mice. After completing an evaluation of the C/F index at 160 days, the RKT diet was replaced with the normal diet. The mice were adapted to the experimental environment for 20 days. Then, the C/F index was evaluated. Columns and bars represent the mean ± S.D. of the C/F index. The data were analyzed for significant differences using one-way analysis of variance (ANOVA), followed by post hoc Bonferroni’s multiple comparison tests. † *p* < 0.0001 vs. 160-day-old SAMP6 mice fed the normal diet. **** *p* < 0.0001 vs. 160-day-old SAMP6 mice fed the RKT diet. There were six to ten mice in each experimental group.

Similarly to SAMP6 mice fed the normal diet, the C/F index in SAMP6 mice fed the RKT diet remained significantly lower than that in SAMR1 mice throughout the entire experimental period (Figure 4). However, the C/F index in SAMP6 mice fed the RKT diet was constantly and significantly higher than that in SAMP6 mice fed the normal diet. The C/F index peaked (49.4 ± 1.4) when the mice were 70 days old and remained mostly constant. The value at 150 days old in SAMP mice fed RKT was similar (48.3 ± 1.2) to that at 70 days old (Figure 5). Interestingly, as shown in Figure 5, we confirmed that the C/F index suddenly decreased on the replacement of the RKT diet with the normal diet within 3 weeks (42.8 ± 0.9). The Pearson correlation found between the two examiners’ who measured the C/F index was 0.9835 (*p* < 0.05).

### 3.4. HUL4 Index of SAMR1 and SAMP6 Mice Fed Diet With or Without RKT

**Figure 6:**
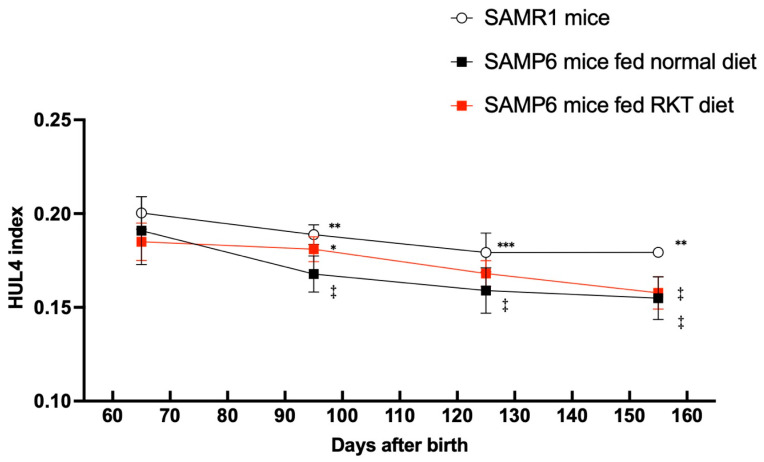
The time-course profile of monthly changes in the HUL4 index of SAMR1 mice, SAMP6 mice fed the normal diet, and SAMP6 mice fed the RKT diet. The X-ray image acquisition was repeated every month from the age of about 70 to 160 days old, and the monthly average of the HUL4 index was analyzed. Points and bars represent the mean ± S.D. of the monthly HUL4 index. The data were analyzed for significant differences using two-way analysis of variance (ANOVA), followed by post hoc Bonferroni’s multiple comparison tests. * *p* < 0.05, ** *p* < 0.01, and *** *p* < 0.001 vs. SAMP6 mice fed the normal diet. ‡ *p* < 0.01 vs. each respective value of 70 days old. There were nine to eleven mice in each experimental group.

As shown in Figure 6, the HUL4 index at the beginning of this study was about 0.192 ± 0.006 in all mice. Similarly to the C/F index, the value in SAMR1 mice remained unchanged during the observation period. Conversely, the value in SAMP6 mice gradually decreased with their growth. The index in SAMP6 mice fed the normal diet was significantly lower than that in SAMP6 mice fed the RKT diet and SAMR1 mice at 100 days. Unlike the C/F index, we found that the HUL4 index in SAMP6 mice fed the RKT diet decreased when mice were 130 days old, and remained unchanged thereafter. The Pearson correlation found between the two examiners who measured the HUL4 index was 0.9997 (*p* < 0.001).

## 4. Discussion

There are many reports on the development of a screening method to detect osteoporosis and osteopenia or a method to estimate BMD from X-ray images obtained from patients [19,20]. We actually reported developing a method to evaluate BMD using clavicle images from a patient’s chest X-ray radiographs, and confirmed that it may be useful for primary osteoporosis screening [10,11]. Recently, BMD estimation using artificial intelligence (AI) or machine-learning (ML) instead of the manual process has been achieved [21,22,23]. According to these technological innovations, AI–ML will be able to automate the opportunistic screening for osteoporosis. However, since we did not obtain X-ray images that were used in the estimation from the same patients, it is considered necessary to obtain sequential and continuous X-ray images from the same individuals in order to understand the long-term series of gradual changes and to comprehend the characteristics of osteoporosis development. There is a small but real risk of long-term effects such as radiation injury, cataracts, or cancer due to increased cumulative radiation exposure with repeated X-ray examination; therefore, we considered that it is necessary to comprehend the changes in cortical bone based on general X-ray images rather than CT or DXA in animals that develop osteoporosis in order to predict the development of osteoporosis before any symptoms become apparent. Previous studies conducted using DXA demonstrated that a decrease in BMD occurs at somewhere between 20 and 24 weeks old (between 140 and 160 days old) in SAMP6 mice [14,24]. We observed that osteogenesis started at the beginning of this study, and the C/F index peaked at around 70 days after birth and persisted for about 3 weeks in SAMP6 mice fed the normal diet; however, their C/F index started to decline, and the value at 140 days after birth had reduced to almost 85% of its peak. The time required to detect the decrease in bone mass using our method was similar to that using DXA. Chen et al. reported that the osteoblast number of the trabecular bone in the femur of SAMP6 mice at 5 months of age significantly decreased without affecting the osteoclast number [14]. Although we did not conduct histological evaluation of the number and activity of osteoblasts and osteoclasts in this study, our results indicated that the decrease in the C/F index is correlated with the reduction in BMD in the femur. However, there was no significant change in the C/F index of SAMR1 mice used as a control group during the observation period. Chen et al. also reported that the number of osteoblasts remained unchanged at 5 months of age [14]. We also found that the C/F index in SAMP6 mice at the beginning of this study was lower than that in SAMR1 mice, and that its peak value in SAMP6 mice was about 80% of that in SAMR1 mice. We also found a similar trend in HU of the L4 vertebral body in SAMR1 and SAMP6 mice fed the normal diet when standardized by the ROI area. However, the decrease in the HUL4 index was observed prior to the decrease in the C/F index in the femur. Silva et al. reported that SAMP6 vertebrae had 33% less trabecular bone volume at 4 months of age (around 110 days old) [25]. It is known that the OVX mouse model has been widely accepted to be suitable for the study of postmenopausal osteoporosis [26]. In our pilot study, we confirmed that the C/F index in OVX female mice was significantly less than that in sham-operated female mice at 6 weeks post-surgery (sham-operated mice: 32.8 ± 2.7, OVX mice: 28.6 ± 0.9, *p* < 0.05). Previous reports indicated that OVX mice rapidly lose metaphyseal cancellous bone within 4–5 weeks [27]. From these findings, we considered that the evaluation for osteoporosis using the C/F index and the HUL4 index may be similar to the results obtained by DXA and histological evaluation.

In general, the gold standard for accurate estimation of BMD is the usage of DXA and X-ray computed tomography (CT) [18]. The effective doses for determining BMD by DXA and quantitative CT in animal studies are typically estimated to be in the range of 20 to 50 μSv and 60 to 100 μSv, respectively [28,29]. The level of radiation exposure from single image acquisition using our system was about 21.0 ± 1 μSv. We also did not note any significant radiation injury, such as weight loss or skin changes, including hair loss, redness, and peeling, at the end of image acquisition. Based on these findings, it is possible that the measurement of the C/F index using our developed method is also useful for assessing bone mass and evaluating osteoporosis and osteopenia in laboratory animals without the need for an expensive device such as DXA or micro-CT. Therefore, it is thought that we should avoid continuous and frequent measurement of the HUL4 index to estimate the bone condition, because the level of radiation exposure from CT image acquisition using our system is about 235 ± 10 μSv.

In this study, we demonstrated that RKT significantly inhibited the decrease in bone mass that accompanies aging in SAMP6 mice. RKT is composed of the following eight crude drug extracts: Atractylodes lancea rhizome, Ginseng, Pinellia Tuber, Poria Sclerotium, Jujube, Citrus Unshiu Peel, Glycyrrhiza, and Ginger. Previous reports showed that several components of RKT regulate ghrelin secretion, ghrelin receptor sensitization, and ghrelin degradation via multiple targets in the stomach and central nervous system [30,31]. Ghrelin can increase gastric-emptying and gastric acid secretion and is a known appetite-stimulating hormone. Based on these findings, RKT has been widely prescribed for patients in Japan with GI symptoms that include loss of appetite, constipation, and GERD [6]. However, there are no reports of RKT improving the condition of bone mass or bone metabolism in human patients. Chiba et al. reported that hesperidin, a flavonoid contained in Citrus Unshiu Peel that is a component of RKT, inhibited bone loss by decreasing the osteoclast number in OVX female mice [8]. Horecajada et al. also demonstrated that the administration of hesperidin inhibited bone loss in OVX rats via an altered nuclear factor kappa B (NF-κB) signaling system, which is an ancient protein transcription factor that controls transcription of DNA, cytokine production, and cell survival, in osteoclast cells [32]. Zhang et al. recently reported that hesperidin attenuated oxidative stress mediators, such as thiobarbituric acid reactive substance, and cytokine levels, such as tumor necrosis factor-α, interleukin-6 (IL-6), and IL-1β, by the inhibition of NF-κB and factor-kappa B, and exhibited a BMD-enhancing effect in OVX rats [33]. Furthermore, previous reports revealed that Ginseng, Poria Sclerotium, and Glycyrrhiza act as bone mass-loss-remedy agents [34,35,36]. It is known that Jujube fruit contains calcium and potassium and supports bone strength [37]. We observed that long-term administration of RKT inhibited the decrease bone loss index in femur, but not in the fourth lumbar vertebral body. Previous reports showed that RKT regulates ghrelin secretion, ghrelin receptor sensitization, and ghrelin degradation via multiple targets in the stomach and central nervous system [30,31]. In our pilot study, we also found that food intake and locomotor activity in SAMP6 mice fed the RKT diet at 140 days after birth was more than those in mice fed the normal diet (daily food intake, normal diet: 4.03 ± 0.06 g, RKT diet: 4.61 ± 0.14 g; daily locomotor activity, normal die: 5554 ± 552 counts, RKT diet: 6857 ± 270 counts, *p* < 0.05.). Based on these findings and our observations, we speculated that the long-term administration of RKT may affect the inhibition of bone loss that accompanies the aging process by direct (bone remodeling) or indirect (ghrelin signaling pathway) modulation. He et al. reported that many kinds of natural medicine except RKT may treat osteoporosis by their the anabolic and anticatabolic effects via the signaling pathway of classical Wnt/β-catenin, TGF-β/Smad, BMP2, Notch, RANKL, MAPK, and NFATc1 families [38]. Further studies are needed to elucidate the role of RKT using this evaluation method by confirming whether RKT itself modifies the function of BMUs, which are the fundamental structures responsible for bone remodeling.

The daily dose of RKT in this study was approximately 1000 mg/kg per unit body weight because the mice typically weighed between 35 and 40 g and ate about 3.5 g of chow pellets with 1% RKT (i.e., daily intake of RKT was approximately 35 mg). However, the human dose of RKT is calculated as approximately 80 mg/kg per unit body weight because the clinical daily dose of RKT is normally 4000 mg, and the average body weight of elderly Japanese individuals is approximately between 50 and 60 kg [39]. Based on these values, the mouse dose was approximately 12.5-fold higher than the human dose; therefore, according to Nair and Jacob’s report [40], the dose of RKT used in the present study is considered to be reasonable and acceptable. These results indicate that RKT can be applied as a preventive medicine for osteoporosis in human patients.

According to Figure 5, the C/F index in SAMP6 mice fed the RKT diet decreased immediately on providing the normal diet instead of the RKT diet. The index value was equal to that in SAMP6 mice fed the normal diet. We also confirmed that the decreased index in SAMP6 could not be restored, even though we provided the RKT diet again. Considering these findings, we suggest that RKT may have preventive but not therapeutic effects on osteoporosis and osteopenia by the inhibition of bone loss. Further experiments will be needed to elucidate the precise mechanism behind the prophylactic action of RKT against osteoporosis and osteopenia in this mouse model and developed evaluation method.

The present study has some limitations. Firstly, we did not confirm the gender differences in the time-course change in the C/F index and HUL4 index. We used only male SAMP6 or SAMR1 mice in this study. Confirmation of their gender differences would have given more strength to this study. Secondly, the C/F index and HUL4 index were obtained manually in a calculation process. This process may be replaceable by AI or ML, which could speed up the process and eliminate errors. Finally, we did not obtain the results by using DXA, micro-CT, or histological evaluation. Furthermore, we did not use a well-established treatment of osteoporosis such as bisphosphonates, RANK ligand inhibitors, sclerostin inhibitor, and parathyroid hormone in this study. Future studies should include these results and confirm the correlation.

## 5. Conclusions

We can readily collect data related to a series of continuous changes in bone mass that accompany aging without the need for an expensive device such as DXA or micro-CT. The repeated administration of RKT resulted in the prevention of decreasing bone mass, indicating the potential of RKT to prevent osteoporosis and osteopenia in human patients.

## Data Availability

The data presented in this study are available on request from the corresponding author. The raw data were generated by equipment installed at Morinomiya University of Medical Sciences. Derived data supporting the findings of this study are available from the corresponding author on request.

## Abbreviations

ANOVA	analysis of variance
BMU	basic multicellular unit
BMD	bone mineral density
C/F index	ratio of cortical thickness of femur
CT	computed tomography
DXA	dual-energy X-ray absorptiometry
GERD	gastroesophageal reflux disease
GI	gastrointestinal
HU	Hounsfield units
IL	interleukin
OVX	ovariectomized
RKT	Rikkunshi-To
ROI	region of interest
SAM	male senescence-accelerated mouse strain
S.D.	standard deviation
